# Displacement of the Uterus, and Some Other Diseases Peculiar to Females, with Cases

**Published:** 1849-10

**Authors:** R. L. Scruggs

**Affiliations:** Germantown, Tennessee


### WESTERN JOURNAL

OF

### MEDICINE AND SURGERY.

---

OCTOBER, 1849.

---

Art. I.—*Displacement of the Uterus, and some other diseases peculiar to females, with Cases.* By R. L. Scruggs, M. D., of Germantown, Tennessee.

Perhaps no part of our extensive country furnishes, for its population, a greater amount or variety of disease than the western district of Tennessee. Its numerous swamps, (particularly in that portion of it bordering on the Mississippi river) traversed by small sluggish streams —its comparatively recent settelement, and generally by poor emigrants from the Atlantic States, who are necessarily compelled to live in indifferent log cabins; and the consequent exposure of both male and female, to a most variable and humid atmosphere, will, I think, sufficiently account for it. Add to this the annual importation, by steamers on the Mississippi, of all the exanthematous and other contagious diseases, with their grave sequelæ, and we have no difficulty in accounting for the amount and variety of diseases generally to be met with here.

This being the case, it is not at all singular that we find diseases of females here, numerous and grave; and it is upon the symptoms and treatment of a few of the most remarkable of these cases that I propose to offer some reflections in this paper.

On the 26th of June, 1848, I visited Mrs. W., æt. 24. She was greatly emaciated; slight cough; tenderness along the course of the spine, and pain in the region of the uterus; countenance expressive of great anxiety and suffering. She had suffered for many months, and was growing worse daily, maugre the attentions of several medical men, to whom it had not probably occurred to make a vaginal examination; or if it had, they had been restrained from asking permission, from ideas of false delicacy, greatly to the injury of the patient and their own reputation. Her nervous system was so seriously affected that she could not converse without shedding tears; and she became so much agitated and wild at times, as to cause serious apprehensions on the part of her husband, that there would be a permanent loss of the mental faculties. With considerable difficulty I obtained her consent to make a vaginal examination, and found the uterus prolapsed and retroverted. I succeeded, without much difficulty, in overcoming the retroversion and placing the organ in its proper position, (for it was neither inflamed nor enlarged)—after which I introduced a glass globe pessary, which retained it in situ until a course of medication could be instituted to relieve the morbid condition of the system, and give tenacity and strength to the debilitated and relaxed uterus and its appendages. To accomplish which, I instituted the following treatment:—directed the spine to be blistered six inches over the dorsal vertebræ, and a stimulating liniment to be rubbed above and below, twice a day, until the blistered surface should heal; then apply the blister below, and rub above with the liniment. At the same time I directed her to take half grain doses of proto. iod. mer., mixed with ext. taraxacum, morning and evening, keeping the bowels gently soluble with any mild aperient. To take five drops of diluted nitric acid (ʒj. nitric acid to ℥j. water) in sweetened water three times a day, gradually increasing the quantity for five or six days, unless some unpleasant effects resulted from it; in which event, the quantity was to be diminished, or the article discontinued for a time; and a solution of iodine and iod. potassium, (iodine grs. x, iod. potas. grs. xx, aqua ℥j) in infusion of sarsaparilla, substituted;—to commence this in ten drop doses, three times a day, gradually increasing to thirty or forty. After pursuing this course for three weeks, I put her upon the preparations of iron, a generous diet, and gentle exercise in the open air. She improved rapidly, and is now in good health.

Another case of displacement of the uterus, differing, however, in many respects from the foregoing, which I had under treatment at the same time, was Mrs. S., æt. 34, the mother of several children. The history of the case, as I learned from herself, was, that about four weeks previous to my visit, in walking from the kitchen to the dining room, with a dish in each hand, her foot slipped, and she fell; to prevent the dishes from being broken she had elevated both hands, making no effort with them to save herself from injury; that she immediately felt severe pain in the "small of the back," went to bed and sent for a physician. Since that time there had been constantly passing from the uterus, in small quantities, a thick dark colored fluid, attended with a great deal of pain. She said that the physician had given her medicines of various kinds, without the slightest benefit. Upon question ing her, I learned that no internal examination had been made, nor any request for permission to make one. The bowels had been irregular, and when hardened feces did pass, it was with pain and difficulty, and were flattened out as if pressed upon by some resisting body. She had become extremely low spirited, and had almost despaired of ever recovering. I told her that I suspected displacement of the womb, and explained to her the necessity of a vaginal examination; to which, after a little hesitation, she yielded. In this examination, the first thing that arrested my attention, was a large, slightly yielding, and somewhat elastic tumor in the recto-vaginal space; so large and peculiar to the touch, that I at first doubted whether it was an extraneous growth, or the fundus of the uterus. I suspected, at first, retroversion, but upon examination, several circumstances were wanting to confirm this diagnosis. The os uteri, instead of being under the symphysis pubis and impinging upon the urethra, was found nearly in its natural position, looking downwards and a little forward, while the cervix seemed to be pushed a little anterior by the tumor behind. This was a little perplexing for a time, but, after a patient examination, I distinctly recognized a displacement that I had never appreciated before, to wit: retroflexion of the body of the uterus. The organ being considerably enlarged and tender to the touch, hypertrophied and inflamed, as I thought, and bowels somewhat constipated, I determined not to attempt its replacement at once, but endeavor to reduce the inflammation and enlargement, if possible, first;—to accomplish which, wet cups were applied over the hypogastrium and sacrum, and an aperient administered at intervals to evacuate the contents of the bowels and keep them soluble. For the hip bath, which I considered desirable, but which could not be used without great pain and inconvenience, warm fomentations were substituted, and used several times a day, for three days. At my next visit, finding the organ in a more favorable condition for the operation, I placed her upon her knees and elbows, introducing the index finger of my left hand into the os tincæ, while with my right hand I passed a sperm candle into the rectum. I thus pressed upon the tumor with the candle, while I manipulated with the left hand, and in this way succeeded in a few minutes in returning the organ to its normal position. She was then put to bed, and requested to remain in horizontal, and, as nearly as possible prone position, until she should be satisfied that she could assume the erect, without displacing the womb.—She was then put upon the proto-iod. mer. in half grain doses twice a day, with inf. senna, to move the bowels when necessary; and a solution of iodide of potassium, commencing with gtts. x, three times a day, in a little sweetened water, and increasing to 40 or 50 drops—solution of the following strength: hydriodate potash grs. xxx, aqua ℥j. The iodide mer. was discontinued in the course of three or four days, but the potash continued to the end of the treatment. She improved handsomely—was entirely well in six weeks—has menstruated regularly ever since, and is now in the enjoyment of perfect health.

M. Velpeau remarks that flexions of the uterus are frequently mistaken for engorgement of that organ;—which latter condition, he thinks, is extremely rare; but I cannot conceive why both conditions should not exist at the same time. Indeed, it seems to me, that a posterior flexion of the uterus is precisely the condition best calculated to bring about this state of things. When we are told, too, that the uterus thus bent upon itself, offers an insurmountable obstacle to the passage of the semen into the cavity of the organ, and in this way causing sterility; we would naturally conclude that the blood vessels were constricted, and, to a certain extent, prevented the return of the venous blood. Add to this the lesion of function that must necessarily exist under such circumstances which, to a certain extent, would prevent an appropriation in the natural way, of the blood sent there periodically; and we must conclude, I think, that unless the organ is replaced very soon after the accident, engorgement will inevitably follow. Whether true hypertrophy exists undr such circumstances, is not altogether so clear.

While on a visit to a patient nine miles distant from my resideuce, in September, 1847, I was requested by Mr. B. (the gentleman at whose house the patient was) to ride with him half a mile out of my way, to see a lady who was supposed to be dying with malignant disease of the uterus. She had requested Mr. B. to bring me to see her at my next visit to his house. I found her in a most deplorable condition indeed, unable, from extreme exhaustion, to assume the sitting posture, without assistance; greatly emaciated, with a dirty yellow tinge of the face; an ugly loose mucous cough, which seemed to jar the whole contents of the thorax; and tenderness at many points along the course of the spine. The stethescope enabled me to discover no abnormal sound in the lungs, except a diffused mucous ràle over nearly their whole extent, which, with the cough, I concluded, might be the effect of the spinal irritation. Examination *per vaginam* revealed nothing abnormal in the position or size of the uterus. Adhering to the hand, however, upon withdrawing it, was an offensive, dirty, yellow looking matter, with small, lumpy and shreddy matters intermixed, somewhat resembling feces. The smell, however was altogether different, and I was perfectly satisfied after the examination that no communication existed between the rectum and vagina or uterus. She informed me that some months before, (the exact time not recollected) the uterus commenced enlarging, the catamenia ceased, and she was under the impression that she was pregnant. The uterus continued to increase, until it attained a certain size; then pains, resembling labor pains, came on, and she discharged a quantity of offensive matter, very much like that which was then passing from her. After this, the matter continued to dribble from her constantly, and she perceived in a short time, that the uterus was again enlarging.—Two weeks from the time of the first discharge, similar pains came on, and a similar discharge from the uterus took place; and this continued to recur every two weeks up to the time I saw her. It was a novel case to me, and without being able to make out the diagnosis to my own satisfaction, I determined to try the virtues of counter-irritants, alteratives and tonics. I directed the spine to be blistered one-half of its length from above downwards and the lower half to be rubbed twice a day with a strong stimulating liniment, and this to be reversed when the blister should heal—blister below and frictions above.—Tartar-emetic pustulations over the chest to be continued until the cough ceased; with an expectorant of lobelia, squills and seneca, to be taken pro re nata. Gave the iod. mer. pill night and morning, and directed her bowels to be moved, when required, with equal parts, by measure, of cremor tartar, magnesia and sulphur. At the same time I directed her to take of the following solution, to wit: ℞ Iod. potassium grs. xx, iodine grs. x, aqua ℥j. Mix. Commencing with 20 drops three times a day, in infusion of sarsaparrilla, and increase gradually to 40 drops—to cleanse the vagina, when necessary, with inf. chamomile. At the expiration of three weeks I learned from her husband that she had greatly improved; the cough having entirely disappeared; no discharge from the vagina, and no appearance of the uterus enlarging. I then directed him to give her mur. tinc. iron in infusion of quassia three times a day, commencing with 10 drops, and increasing to 25 or 30—to be alternated at intervals of five or six days, with the nitric acid lemonade.

Five weeks from the time of my first visit, I called to see her, and was surprised and delighted to find her apparently well, walking about and attending to her domestic duties. She rapidly gained flesh and strength, until her health was perfectly re-established. She informed me some time after, that her catamenia continued to return with perfect regularity; and that the quantity, color, &c., evinced a perfectly healthy condition of that function.

The history of this case, as given by the lady herself, up to the time I first saw her, is incomplete, and may possibly, in part, be incorrect. With regard to the exact periodicity in the efforts of the womb to expel its contents, I have some doubts; but as to the fact of the matters accumulating there, and being expelled in the manner described, I have none whatever. I omitted to mention, in its proper place, that the morbid contents of the uterus had been expelled the day before I first visited her (amounting to more than two quarts), and never afterwards re-accumulated. Was this a case of simple chronic inflammation of the lining membrane of the uterus? producing the, so called, hydrometra? or was it malignant disease of that organ?

In the latter part of May, 1847, I saw, for the first time, Mrs. N., æt. 30, the mother of three children. She had suffered for many months with irregularity in the catamenial efforts, and general derangement of all the functions of the body, producing great emaciation, a long train of hysterical symptoms, &c. Her bowels had been much disordered during the time, and continued to get worse, under the treatment of a "steam doctor," until, finally, a severe form of chronic dysentery was the result. Examination *per vaginam* disclosed prolapsus and a most singularly atrophied condition of the uterus. Indeed, it was impossible for me, with the most careful examination, to detect anything of the organ, except the cervix, which the extremely relaxed condition of the walls of the vagina permitted to be moved in any direction with great facility.

I will not go into the details of the treatment in this case, but content myself with simply submitting the general plan, which was similar to that instituted in the first case, (Mrs. W.) i. e. the same articles were brought into requisition, and varied in their administration according as circumstances seemed to indicate. Proto-iod. mer., hydriodate potas., nitric acid, elix. vitriol, cit. and mur. tinc. iron, were all used—first one and then the other, in the order in which they are mentioned, each one continued as long as the patient seemed to be benefitted by its use; substituting another article and again returning to the one that had been previously given.

This plan may, at first view, be considered empirical, but upon a little reflection, it will be remembered, that it often happens, that while one particular article of the materia medica, will, for a time, act most beneficially, filling the indication admirably; it appears at last to loose its powers, and may be advantageously superseded by another of the same class, and again recurred to at a later period of the disease. At any rate, this plan, in my practice, has answered all my expectations, and I shall continue to pursue it, until I am satisfied of its inutility. It answered an admirable purpose in this instance, making a perfect cure at last, but the time required to accomplish it was much greater than in either of the preceding cases mentioned in this paper. I saw her on the 10th of last January, and learned that she had been delivered, six weeks before, of a healthy child, weighing, at its birth, eleven pounds. Mother and child both doing well at the time I saw them.

In addition to the treatment already mentioned, the bowel affection was attended to in the following manner, to wit: dry cups, stimulating frictions and the flannel roller over the abdomen—internally, a combination of hydr. c. creta, Dover's powder, pulv. g. Arabic and nut galls; and also, occasionally, a pill of opium and sulph. cupri, ext. hyosciamus and morphia, to allay nervous irritability and procure sleep. Quinine was used late in the treatment, in small doses, (gr. j.) with the happiest effects. A rather distressing pruritus and leucorrhea, which supervened in the course of the disease, yielded quickly to injections of acet. plumbi.

Germantown, Tenn., Aug. 9, 1849.

